# Impact of COVID-19 on Continuing Medical Education—Results of an Online Survey Among Users of a Non-profit Multi-Specialty Live Online Education Platform

**DOI:** 10.3389/fmed.2021.773806

**Published:** 2021-11-15

**Authors:** Tobias L. Schulte, Thilo Gröning, Babett Ramsauer, Jörg Weimann, Martin Pin, Karen Jerusalem, Sami Ridwan

**Affiliations:** ^1^Department of Orthopedics and Trauma Surgery, St. Josef-Hospital, Ruhr-University Bochum, Bochum, Germany; ^2^Joint Practice for Gynecology, Moenchengladbach, Germany; ^3^Department of Gynecology and Obstetrics, Vivantes Klinikum Berlin, Berlin, Germany; ^4^Department of Anesthesiology and Interdisciplinary Intensive Care Medicine, Sankt Gertrauden Hospital, Berlin, Germany; ^5^Emergency Department, Florence-Nightingale Hospital, Duesseldorf, Germany; ^6^German Society of Emergency and Acute Medicine DGINA, Berlin, Germany; ^7^Department of Neurosurgery, Klinikum Ibbenbueren, Ibbenbueren, Germany

**Keywords:** COVID-19, continuing medical education, online education, medical education, survey, online teaching

## Abstract

**Background:** The Coronavirus Disease-2019 (COVID-19) pandemic accelerated digitalization in medical education. Continuing medical education (CME) as a substantial component of this system was relevantly affected. Here, we present the results of an online survey highlighting the impact on and the role of online CME.

**Methods:** An online survey of 44 questions was completed by users of a German online CME platform receiving an invitation via newsletter. CME habits, requirements, personal perception, and impact of the pandemic were inquired. Standard statistical methods were applied.

**Results:** A total of 2,961 responders took the survey with 2,949 completed surveys included in the final analysis. Most contributions originated from Germany, Austria, and Switzerland. Physicians accounted for 78.3% (57.5% hospital doctors) of responses followed by midwives (7.3%) and paramedics (5.7%). Participating physicians were mainly board-certified specialists (69%; 55.75% hospital specialists, 13.25% specialists in private practice). Frequent online lectures at regular intervals (77.8%) and combined face-to-face and online CME (55.9%) were favored. A duration of 1–2 h was found ideal (57.5%). Technical issues were less a major concern since the pandemic.

**Conclusion:** A shift from face-to-face toward online CME events was expectedly detected since the outbreak. Online CME was accelerated and promoted by the pandemic. According to the perception of users, the CME system appears to have reacted adequately to meet their demand but does not replace human interaction.

## Introduction

Digitalization in healthcare had been pursued for the last decade and lies mainly in the hands of governments and healthcare systems (1). Digitalization has obviously moved into focus around the globe since the Coronavirus Disease-2019 (COVID-19) pandemic. Medical education also had to adapt to the implications of the pandemic and corresponding political measures. Prior to the outbreak of COVID-19, digital medical education was some kind of modern luxury and technical achievement in selected countries. Due to COVID-19, the digitalization of medical education was forced to move forward at an unprecedented pace, developing from a nice-to-have luxury to an absolutely essential tool (2–9).

No doubt, that the pandemic has raised the value of such platforms and educational formats, as physical presence is not required. Not only board-certified specialists but also trainees and medical students had to abandon well-established and familiar educational practices (10). Teachers and students, lecturers, and attendees were confronted by these facts similarly. The impact of the pandemic on in-hospital medical training and CME has already been picked up by recent literature, highlighting the processes and possibilities at hand (4, 9, 11).

MEDIZIN TO GO is a free of charge German continuing medical education (CME) platform offering online multi-specialty live educational lectures with approved medical certification and medical board CME accreditation since 2012 (Table 1). Thus, not only does it offer online medical lectures on relevant and up-to-date topics but also medical professionals are given the opportunity to obtain required continuing medical certification relevant for their practice without having to attend face-to-face lectures and conferences. Notably, a total of 250 CME accreditation points are mandatory for German board-certified physicians every 5 years. The main goal of the platform is to adequately prepare residents for board exams. However, the format also attracts medical students, experienced physicians, and related healthcare professions, such as midwives, nurses, physical therapists, or paramedics. MEDIZIN TO GO is independent of any industrial influence, medical society, or other stakeholders.

**Table 1:** Online continuing medical education on MEDIZIN TO GO (November 2020).

**Format**	**Specialty**	**Online Since**	**Online Events/Year**	**Event Duration**	**Additional events**
GYN TO GO	Gynecology, Obstetrics, Endocrinology and Senology	09/2011	45 early morning events* and 45 late night events + additional events	45 min.	4–5 Weekend events*/year (4 h)
NOW TO GO	Emergency Medicine	01/2017	45 early morning events and 45 late night events + additional events	45 min.	4–5 Weekend events/year (3 h)
OU TO GO	Orthopaedics and Trauma Surgery	03/2020	48 early morning events and 48 late night events	45 min.	No
PAED TO GO	Pediatrics	10/2020	24 late night events + additional events	50 min.	Weekend Events
AINS TO GO	Anesthesiology, Intensive Care Medicine, Emergency and Pain Medicine	03/2020	11 events	3 hrs.	No
NCH TO GO	Neurosurgery	09/2020	21 late-night events + additional events	45 min.	Weekend Events

*Distribution of currently active medical specialties with past online CME live events on MEDIZIN TO GO at the time the survey was conducted. Other specialties only offering live events after the survey was conducted are not listed*.

* *Online live lectures consisting of a 45-min lecture, with a subsequent live discussion round. Weekend events consisted of multiple lectures related to a selected main topic with live discussion. GYN TO GO, NOW TO GO, OU TO GO, PAED TO GO, and NCH TO GO offer this type of CME. AINS TO GO events are similar to formerly described weekend events. CME certification by the corresponding German medical board requires 45-min events for one CME accreditation point*.

Continuing medical education plays a major role in maintaining up-to-date medical care. With CME being the sole mandatory source of education for German physicians after board certification, a significant impact of the pandemic and precautionary measures, such as social distancing, was to be expected. Being active since 2012, MEDIZIN TO GO with its wide reach (over 20,000 registered users) offered the possibility to analyze the perception of users concerning online CME in general during a broad time window, in addition to changes determined by the pandemic.

To better understand the specific impact of the pandemic on German CME, we conducted a platform-wide online survey. This survey investigated the role of online CME among German-speaking healthcare professionals. Additional focus was laid upon the impact of the COVID-19 pandemic on the perception of participants of CME in general and the online form in particular. Furthermore, the expectations and requirements of the participants regarding online medical education, in general, were highlighted. One of the main concerns of the authors was not only how to further improve CME especially adapted to the COVID-19 pandemic but also for the return of peacetime. Several publications described the impact of COVID-19 on medical education (2, 9, 12, 13); however, only very few formats or platforms allow a direct comparison before and after the onset of the pandemic. Only few published data on this topic exist for the German healthcare system (11, 14, 15).

## Methods

### Study Setup, Survey Design, Validation, and Distribution

A questionnaire consisting of 44 questions was drafted to assess many aspects of online continuing medical education among German-speaking medical professionals and to further evaluate the impact of the COVID-19 pandemic (In the German language; translated version of the survey can be found as a Supplementary Material). The research goal was to investigate and record the impact of the first year of the pandemic on online continuing medical education through the acquisition of qualitative data.

Two experienced academic healthcare professionals with prior experience with healthcare surveys and online CME [SR (16) and TS] arranged the initial draft of the survey. KJ with a degree in education (Diplom Pädagogin) undertook further improvements and assisted with the final version of the survey. The design and internal validation stage was conducted during the second half of November 2020 in accordance with the existing literature (17, 18). A face validity index (FVI) of at least 0.83 was considered acceptable (19). External validation was conducted in a two-step approach. Pretesting was performed on a small sample of board-certified healthcare professionals (*N* = 12) followed by pilot testing on a larger cohort. The calculated S-FVIs were 0.98 and 0.91 based on the average method (S-FVI/Ave) and the universal agreement method (S-FVI/UA), respectively. The estimated completion rate according to SurveyMonkey was at 62%. Given that recently published surveys among healthcare professionals presented response rates between 3 and 5% when calculated using the distribution platforms (excluding social media) and had completion rates at about 70%, this study was intended to reach at least comparable response and completion rates (20, 21).

The final questionnaire was prepared and distributed using the online platform SurveyMonkey (https://www.surveymonkey.com, SurveyMonkey Inc., San Mateo, CA, USA) and was opened to responders on December 1, 2020. The survey was promoted during online lectures, and invitations were sent via E-mail to all 21,007 members of the platform with a newsletter subscription (the targeted population; sample frame *N* = 21,007). Additionally, a web link to the survey was continuously displayed on the website of the platforms (News) for a total of 5 consecutive weeks. Participants receiving the survey were encouraged to inform colleagues by disseminating the web link via social media and other means of interpersonal communication. Data evaluation was started on January 17, 2021.

### General Educational and Demographic Data

Responders were asked to provide information regarding their age, gender, country of origin, and the medical profession. Type of practice (e.g., hospital and private practice) and level of education/experience were inquired in physicians. Sources of CME, number of face-to-face events per year, concerns regarding online education, having attended online sessions, and at what frequency per year were surveyed to better assess the role of online CME. Furthermore, the importance of non-profit sponsoring free and free of charge education, the importance of active participation in discussions, anonymity, video functions, duration of each session, and further technical issues were inquired.

Participants were also requested to describe the ideal form of education and corresponding characteristics within the margins of multiple-choice and numeric scale answers. Additional free-text answers and comments were allowed to better display the improvement suggestions of responders beyond the rigid margins of questions with distinct answer options.

### Online Continuing Medical Education and COVID-19

Besides general educational and demographic data, the questionnaire was intended to capture the impact of the pandemic on the educational behavior of responders. The number of face-to-face and online educational sessions per year was inquired before and since the pandemic to better understand the demand for online lectures. This is related to online medical education *per se* and to this specific platform. Moreover, concerns about using online services before and since the pandemic.

#### Platform Specific Questions

A part of the survey was designed to allow members of the platform with previous experience with its online services to provide anonymous feedback and evaluate technical and content-related issues. Corresponding data were excluded from analysis, as these do not add relevant value to this study.

### Data Analysis

Anonymized data analysis was performed utilizing SPSS 25.0 for Mac (IBM Corp. Released 2017. IBM SPSS Statistics for Mac, Version 25.0. Armonk, NY: IBM Corp.). Prior to data analysis, free text passages were thoroughly reviewed for typing and form errors with possible impact on software analysis and were properly aligned. Numerical scale answers (0–100 and 0–10) were used to identify four groups of responders depending on the individual perception of the issues investigated in this survey and listed above. As such, groups were classified as minor (<25 or <2.5), low intermediate (25 to <50 or 2.5 to <5), high intermediate (50 to <75 or 5 to <7.5), and major (75–100 or 7.5–10) for any aspect investigated.

Univariate analysis was performed to identify possible significant differences, when applicable. The Fisher exact test and the chi-square test were used to analyze categorical variables, and the Student *t*-test to analyze continuous variables. A power analysis was not required, due to the descriptive character of the survey without pursuing a specific hypothesis.

### Ethical Approval and Data Protection

The study design was conducted in accordance with the declaration of Helsinki. Ethical review and approval were not required for this study in accordance with the local legislation and institutional requirements. The survey included a preliminary introduction regarding the nature of the study and an opt-out option asking to formally agree with the participation in this survey. Data protection/privacy policy was clearly provided by the survey platform, it applies to all data recorded using this survey (https://www.surveymonkey.com/mp/legal/privacy-policy/). The study adhered to the 2016 version of the General Data Protection Regulation (GDPR) applicable in Europe since 2018 (https://gdpr-info.eu/). Data with potential personal data protection risk were planned to be deleted from all files and platforms after the final data analysis.

The translated full-version questionnaire, such as multiple choice and free text options, is shown in Supplementary Data.

## Results

### Participating Healthcare Professionals

Two thousand nine hundred and sixty-one questionnaires were returned, 2,949 were properly completed and included in the final analysis. Responders from the three main German-speaking countries (Germany, Switzerland, and Austria), the Netherlands, other European countries, and from outside Europe contributed to the partially global outreach of this project, which was mainly aimed to reach German-speaking healthcare professionals (Survey in the German language). A completion rate of 99.6% (2,949/2,961) was realized, as almost every initiated survey was adequately completed. As some questions were only intended for specific user groups, being not filled out by other groups was not acknowledged as an incompletion. The estimated completion rate calculated by the software was 62%. A response rate could not be accurately calculated based on the method of distribution utilized for this survey also including social media. In light of formerly performed worldwide surveys among healthcare professionals, the response rate for this survey was slightly higher at 14% (2,961/21,007 newsletter subscribers) (20, 21).

**Figure 1:**
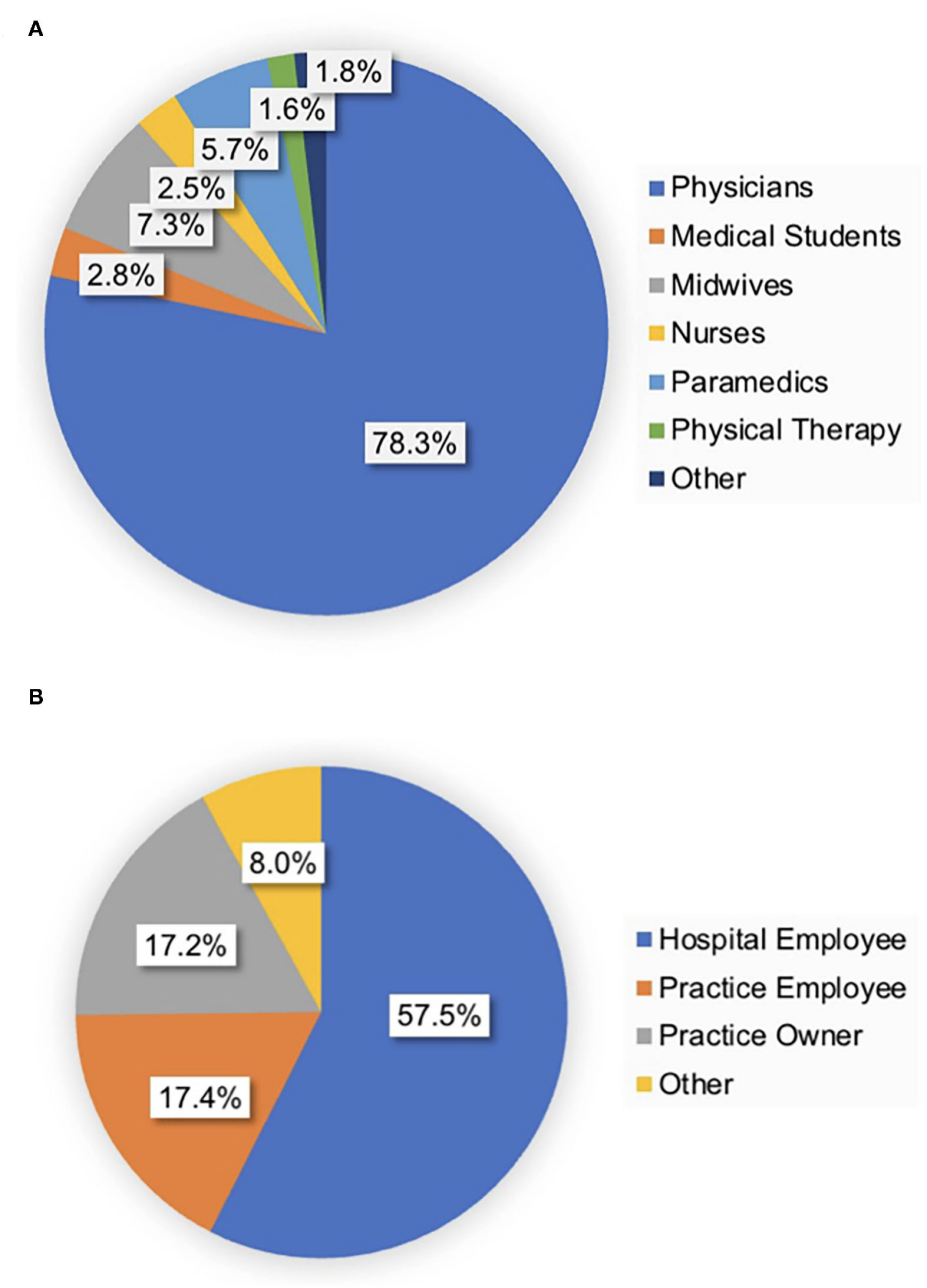
Healthcare professions of participants and occupation of physicians. **(A)** The majority of responders were physicians (78.3%) followed by midwives and paramedics. **(B)** Most physicians were hospital employees.

Most contributions originated from Germany, followed by Austria, Switzerland, and the Netherlands (86.88, 3.39, 1.63, and 0.20%). Responders from other European countries and countries outside Europe accounted for 4.24 and 3.66% of answered surveys. The median age was 40 years (range 17–80). Total 71.4% of responders were female healthcare personnel (27.8% male and 0.1% diverse). Physicians accounted for 78.3% (57.5% hospital doctors, 17.3% private practice employees, 17.2% private practice owners, and 8.0% other employments) of responses followed by midwives (7.3%) and paramedics (5.7%). On closer analysis, participating physicians were mainly board-certified specialists (in total 69% of responding doctors; 55.7% hospital specialists, 13.3% specialists in private practice). Total 3.2% were department heads, 17.2% attending specialists/consultants, and 31% residents/trainees. Figure 1 demonstrates the distribution of healthcare professions.

### Continuing Medical Education and COVID-19

**Figure 2:**
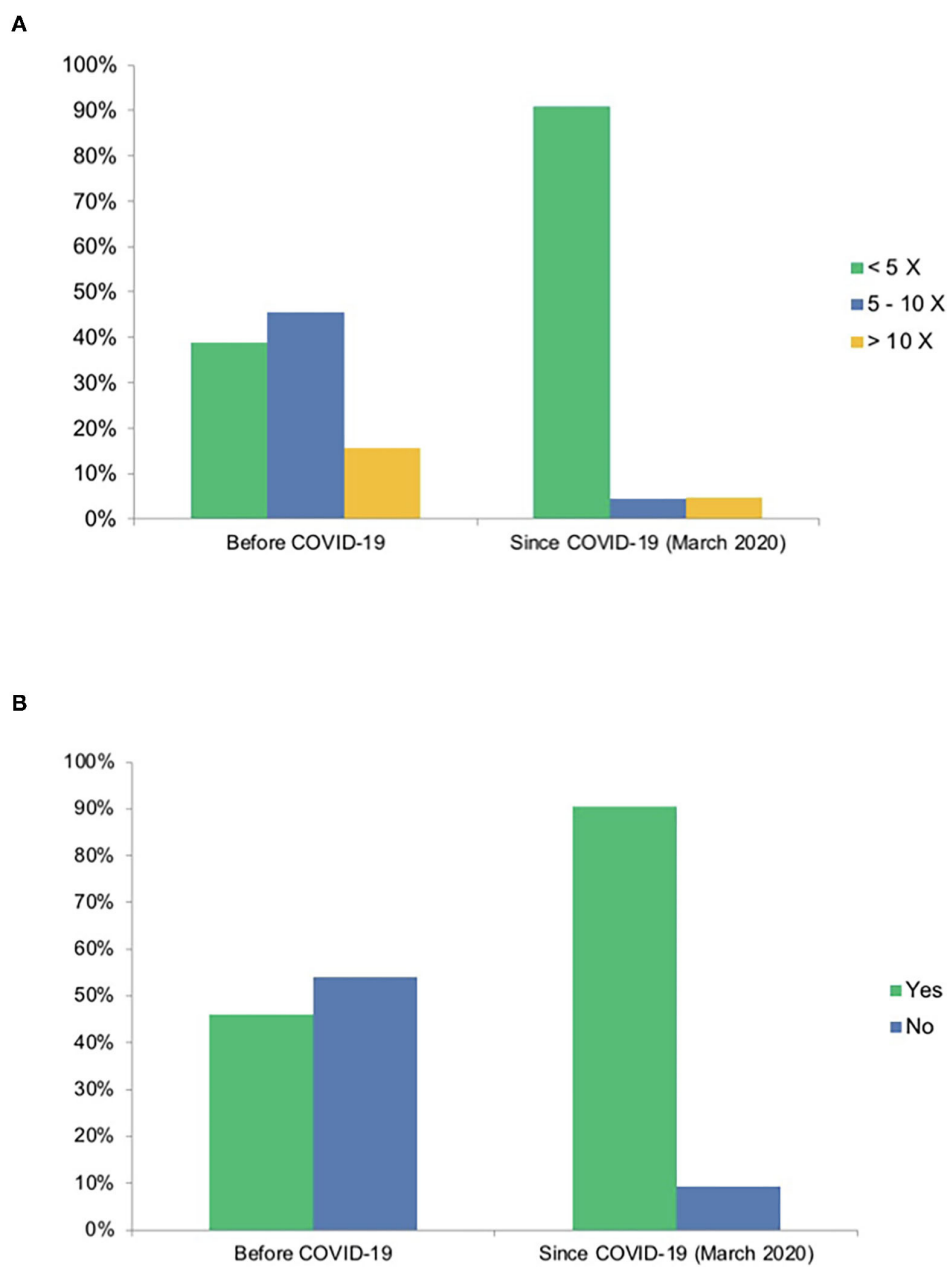
Reported shift in medical education due to COVID-19. **(A)** Number of attended face-to-face events (percentage). **(B)** Attendance of online events (percentage). **(A)** A shift away from face-to-face events for continuing medical education was expectedly detected since the outbreak. **(B)** Also, a shift toward online events can be documented.

**Figure 3:**
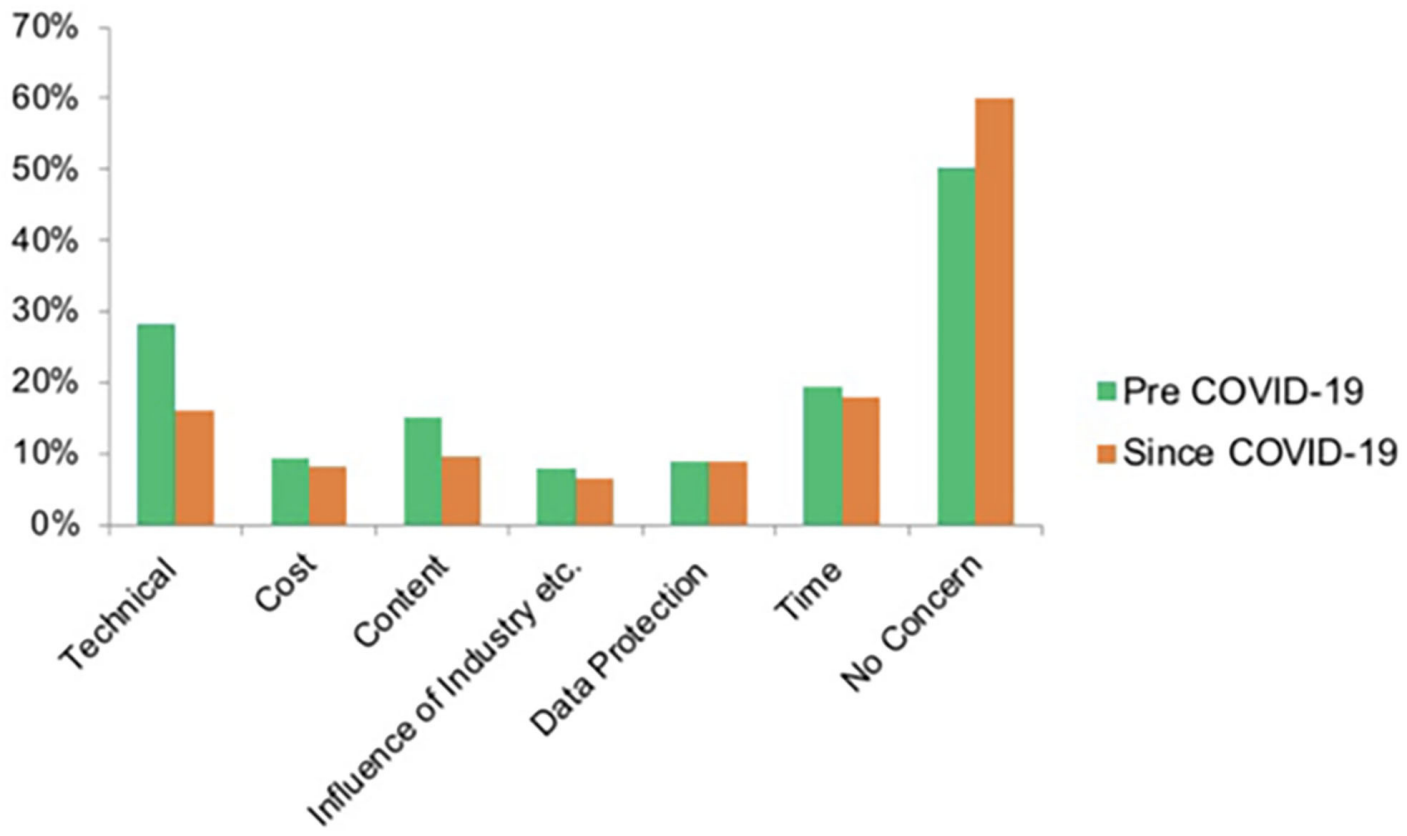
Concerns of responders regarding online CME.

A relevant part of the survey was intended to highlight the impact of the pandemic on CME, online CME in particular. When asked about the number of yearly visited face-to-face CME events, a shift toward online CME was clearly visible (Figure 2). When only 38.9% of responders stated visiting <5 face-to-face events per year before, 90.8% reported doing so since the pandemic, and 46% reported using online CME before compared to 91% since COVID-19. On a closer analysis, 87.3% reported using online CME more frequently since the pandemic compared to 1.6% less frequently and 11.1% unchanged. Overall, 87.8% of participants used MEDIZIN TO GO for online CME since COVID-19 (60.5% before). Analyzing the number of visited online CME events, 35.3% visited 5–10 or >10 before, opposed to 75.1% since the outbreak (43.6 vs. 68.1% for MEDIZIN TO GO). Major concerns regarding online CME before and since the pandemic were also inquired. More than half the participants stated no concerns either before or since the pandemic (50.1 and 60%). Major concerns identified by participants were time and technical issues (19.5 and 28.3% vs. 18.0 and 16.1%). Therefore, technical issues seemed to be less of a problem since the outbreak (Figure 3).

The majority of participants stated having no concerns regarding online CME before and since the pandemic. Major concerns identified by the survey were technical and time issues. Technical issues seem to have declined since the pandemic.

### General Perception of Respondents of (Online) Continuing Medical Education

**Figure 4:**
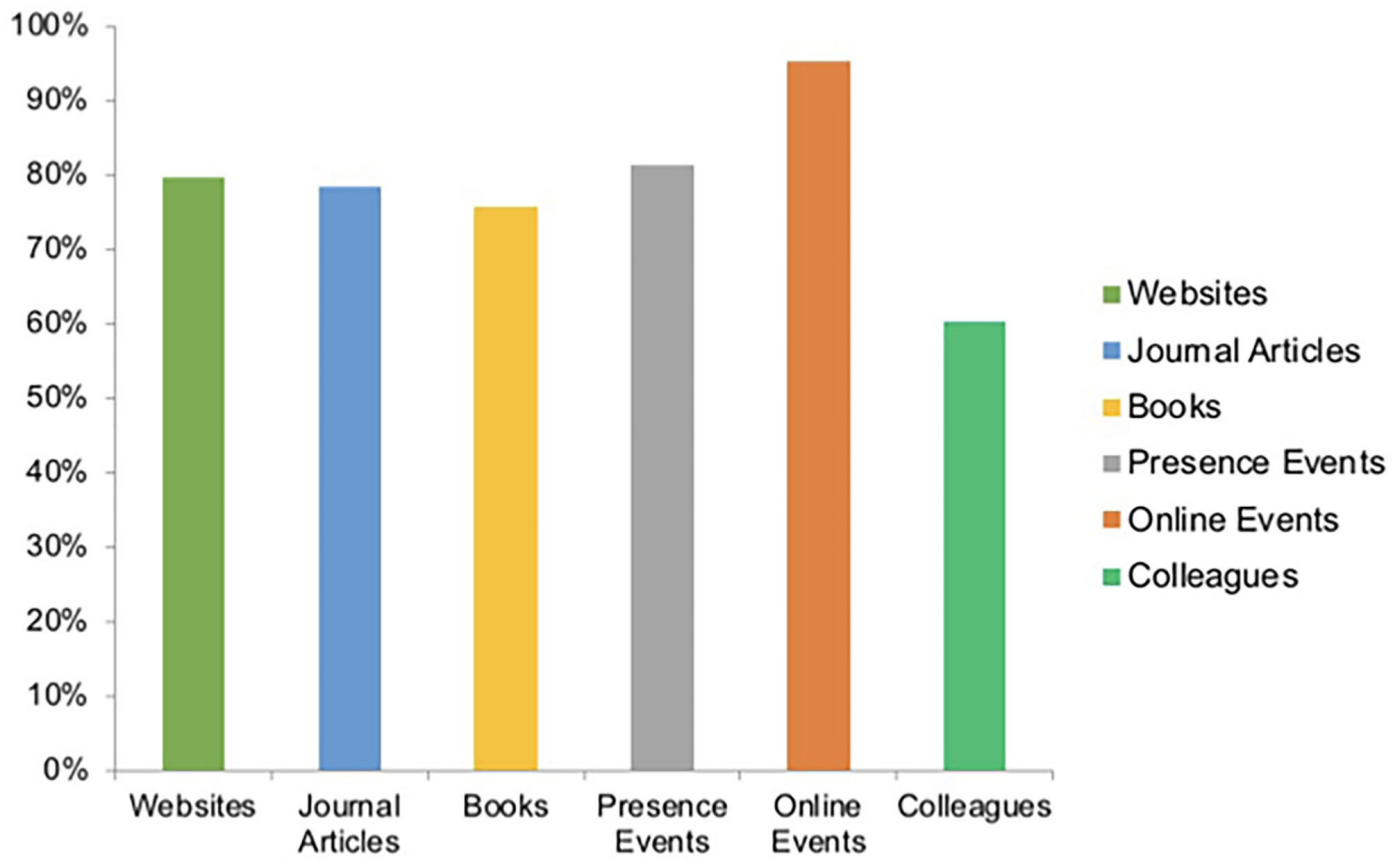
Sources of continuing medical education (percentage). Most responses identified online and face-to-face events as the main source of continuing medical education. Percentages were calculated for a question with multiple answer options.

Participants identified online and face-to-face lectures as the main source of continuing education. However, journals, websites, books, and colleagues were also chosen as corresponding educational references (Figure 4). Free-text answers also named apps, guidelines, departmental journal clubs, and podcasts were considered as additional means of education. Participants were in favor of frequent online lectures at regular intervals (77.8%) and combined face-to-face and online educational formats also known as blended learning (55.9%). In addition, lectures followed by discussion rounds (48.3%) or combined lectures with expert discussions (43.5%) were identified as more suitable for online lectures than sole expert discussions (8.2%). Responding professionals found a duration of 1–2 h ideal (57.5%) compared to <1 h or 2–3 h (40.1 and 2.4%). Evening sessions were generally preferred by 73.1% of answers (15.6% morning and 11.3% weekend) and most attended online CME by oneself (83.6%; 4.8% in a group, 11.6% both).

**Figure 5:**
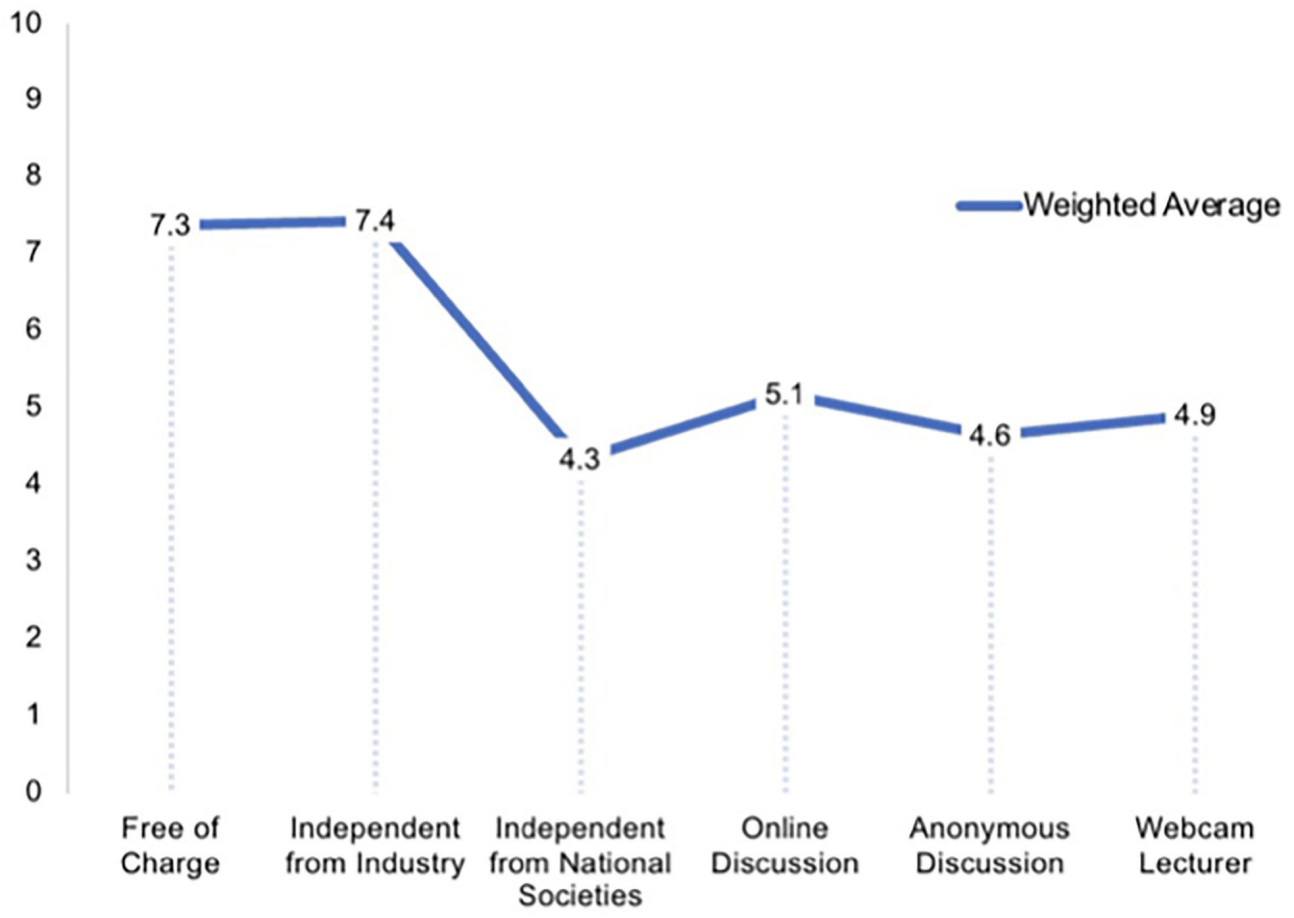
Expectations of participants of continuing medical education. Participants strongly prefer continuing medical education to be free of charge and independent from industrial influence. Other content-related issues were less relevant.

To further assess how ideal online CME should be composed, the survey offered numeric scale questions (0 as not important-−10 as most important) to evaluate the importance of specific composition and content-related parameters. Free of charge and sponsoring free (i.e., not related or sponsored by the Industry) online CME was of high intermediate importance for users (average points on 0–10 scale: 7.4 ± 2.5/10 and 7.4 ± 2.9/10). Being independent of national medical societies was less an issue (4.3 ± 3.1/10). Having a possibility for live discussions after lectures were also of high intermediate (5.1 ± 2.9/10) interest. Interestingly, it was reported to be even less important to be able to discuss anonymously (4.6 ± 3.2/10). The lecturer being visible by webcam was also only of low intermediate relevance (4.9 ± 3.3/10; Figure 5). Participants were inquired to rate the relevance of online CME content-related specifications. Basic clinical knowledge in form of a structured curriculum and content being evidence based was of high intermediate value (7.1 ± 2.5/10 and 6.8 ± 2.5/10). Special and advanced content seemed more important than basic content (7.8 ± 1.9/10). Online CME being some kind of an expert discussion round/meet the experts was of lower interest but remained high intermediate (5.7 ± 2.5/10). Attending online CME in form of online conferences or congresses was of comparable high intermediate interest (5.7 ± 3.0/10). Participants were satisfied with MEDIZIN TO GO as a CME platform at an average of 88% (average percentage on 0–100% scale: 0 not satisfied at all-−100% fully satisfied).

The certification process to acquire CME points (in Germany obligatory for specialist physicians; 250 points per 5 years) was reported to be utilized in 74% of responses (note that 78.3% of participants were physicians).

Overall, the majority of participants would recommend online CME (9.2 ± 1.4/10). To further capture the respondents' expectation of future CME (i.e., after the pandemic), two specific questions on this topic were inquired. Total 87% of participants would prefer the availability of online CME to be similar to COVID times, while only 13% stated that it should be less available after the pandemic. When asked what respondents would rather attend for CME, 30% picked only online, 14% only face-to-face, and 56% a combination of both face-to-face and online CME formats.

## Discussion

The COVID-19 pandemic has forced medical societies and educational institutions to develop and implement new strategies to minimize close inter-personal contacts. This has substantially supported digital online education. This survey addressed the perception of participants of past and present CME; online CME, in particular, inquired expectations of future CME and offered a somewhat realistic presentation of the impact of the pandemic on CME.

### Pros and Contras of Online (Continuing Medical) Education in General

The obvious advantages of online education are being easy to access without requiring physical attendance. Lecturers are therefore able to contribute and present their content globally. Thus, making lectures available for attendees who otherwise would only be able to take part having to travel far distances. One major advantage during the pandemic was and still is limiting the risk of being infected.

The disadvantages of online courses on the other hand are the lack of direct personal interaction. In cases of large groups, face-to-face interaction during lectures is an important feedback tool for both lecturer and trainee. Without, valuable communication is unfortunately lost. In addition, online education carries the risk of diminishing discussions with peers. There is a substantial psychological impact of isolation. Online platforms cannot provide direct interaction with a patient and are highly dependent on technical requirements. The issue of licensing and credits in online education is yet to be uniformly defined. The lack of hands-on activities in sole online educational events is a relevant problem and makes a combination of traditional face-to-face learning with online formats (Blended learning) a necessity. However, several digital surgical skill-training resources are on their way from development to everyday tools, such as virtual reality trainers, simulation models, video games, surgical videos, and smartphone applications (22).

### Results of The Multinational Survey

#### General and Demographic Data

The relevance of this topic was clearly highlighted by the number of participants and the high completion rate. With a total of 2,949 completed surveys included in the final analysis, this study represents the largest pool of responders (In the German language) related to this matter until this day. The age of participants (median 40 years) adequately represented the targeted group. The range (17–80 years) also demonstrated that very young (to be) and elderly (retired) healthcare professionals could be reached by this survey and relate to online CME. Given that physicians require CME certification on a regular basis (in Germany 250 CME points per 5 years), the main group of responders was physicians (78%). Notably, midwives and paramedics each contributed to more than 5% of participations (7.3 and 5.7%), also demonstrating the profession-independent relevance of CME. Female healthcare professionals provided over 70% of responses. This might be explained by the fact that gynecology and obstetrics (GYN TO GO) had been established over 10 years ago and attracts almost 2,000 attendees per live online lecture today. The high percentage of board-certified physicians can be related to CME certification being obligatory for specialists (voluntary for trainees/residents) at least in Germany but might also underline the quality and clinical relevance of the offered online CME lectures.

#### COVID-19 and Online CME

A major part of the composition of the survey was intended to detect the impact of COVID-19 on CME in general and the view of the participants of and attitude toward online CME in particular. The expected shift away from face-to-face toward online CME could be distinctly observed and is mainly a result of social distancing, political measures and infection risk minimization. This was detected in general and specifically for MEDIZIN TO GO as a platform. A relevant subjective concern of both participants and lecturers regarding online CME was of technical nature. On the one hand, this should raise interest in improving technical knowledge in both groups. On the other hand, the results of our study showed that before the pandemic technical concerns had been evaluated much higher than during the pandemic (Figure 3). This might mirror users having realized that technical boundaries are not as present as they expected them to be. In addition, this might represent the fast technical advancement probably resulting in easier access and user-friendly applications.

#### Formats Like MEDIZIN TO GO and The Future of CME

Finding a suitable solution to keep medical education up and running during the pandemic is one challenge. However, an even greater one is how to setup education after the pandemic. Will continuing medical education return to old habits? It might be possible to develop and adopt novel educational systems reasonable during the pandemic but also in the long run afterward, especially as it is realistic that pandemics might repeatedly occur in the future and that recipients of CME might have settled with current possibilities or even prefer the change (2). The current wave of digitalization shall push medical education into a real digital transformation (11, 14, 15, 23). The current crisis should be recognized as an opportunity for medical education to permanently adopt and implement digitalization using modern online formats and maybe combined (Blended learning) events after the pandemic. Of course, online courses cannot and should not generally replace face-to-face in-classroom teaching. Both formats can perfectly complement each other for better results. Participants clearly highlighted the importance of evidence-based content, free of charge education, having the possibility for live discussion, and that they would preferably attend events reoccurring on a regular basis rather than sole sporadic lectures and also combined face-to-face and online events rather than one single format (56% of responses). The demand for online CME events was obviously demonstrated by the survey, also with regard to The Future of CME. Total 87% of participants expect the availability of online CME to remain as high after COVID-19. Online courses as offered by MEDIZIN TO GO are held live, free of charge, non-profit, and are independent of industrial influence (Table 1). Due to the simple and easy access, standard technical requirements, and open number of participants, such platforms offer a virtual lecture hall for live lectures utilizing well-known lecturers and reaching attendees independent from physical, regional, and financial boundaries. In addition, discussion can be joined anonymously, allowing reluctant attendees to actively participate. The live character with room for live discussions is a substantial advantage compared to widespread-recorded online educational material and is one step closer to real human interaction but finally cannot replace it.

### Limitations

The results of this survey are limited by the number of participants, their medical profession, level of education, and intentionally by the subjective statements of single individuals. While the majority of questions were designed as multiple choice, the free text could be submitted in various subsections of the survey hence the total amount of information provided might be hardly comparable. Although the study design focused on the potential for the data collected to be representative, answers are subject to bias. Since more than two thousand German-speaking healthcare professionals and medical students participated, this survey is subject to individual variations among participants, their personal circumstances, working and educational routine and individual aversion to uncertainty might have heavily influenced their answers. However, it has to be considered that in pragmatic qualitative studies with an inductive approach (exploring the characteristics of a problem, not of the subjects), representativeness is not the priority (24). This was precisely the intention of this survey: simply to document the perceptions of CME pre- and during COVID-19 among users of a single online platform (MEDIZIN TO GO) and to help improve CME for upcoming generations and for post-COVID-19.

## Conclusion

Continuing medical education is a major part of healthcare and medical education. Digitalization was accelerated by the COVID-19 pandemic. Fortunately, digital and online CME platforms already existed and bridged the gap until some kind of universal adaptation settled and adequate online CME became abundant. Users have specific requirements, value human interaction, and are in favor of combined face-to-face and online CME events (Blended learning) defining the way for and The Future of CME development.

## Data Availability Statement

The raw data supporting the conclusions of this article will be made available by the authors, without undue reservation.

## Author Contributions

TS and SR devised the project, the main conceptual ideas, the proof outline, supervised and finalized the manuscript, and performed an independent literature search. SR carried out data acquisition, worked out almost all of the technical details, performed the statistical calculations, and drafted the manuscript assisted by TS. TG, BR, JW, MP, and KJ contributed to the survey draft, carried out additional data assessment, and assisted with manuscript completion and the submitted figures. All authors contributed to the article and approved the submitted version.

## Funding

The SurveyMonkey license was privately funded by SR.

## Conflict of Interest

The authors declare that the research was conducted in the absence of any commercial or financial relationships that could be construed as a potential conflict of interest.

## Publisher's Note

All claims expressed in this article are solely those of the authors and do not necessarily represent those of their affiliated organizations, or those of the publisher, the editors and the reviewers. Any product that may be evaluated in this article, or claim that may be made by its manufacturer, is not guaranteed or endorsed by the publisher.
