# Supplementary material for: Impact of COVID-19 on Continuing Medical Education—Results of an Online Survey Among Users of a Non-profit Multi-Specialty Live Online Education Platform
Source: Front Med (Lausanne). 2021 Nov 15;8:773806. doi: 10.3389/fmed.2021.773806 (PMC8634132; doi:10.3389/fmed.2021.773806)
Supplement: Supplementary file 1 [file Data_Sheet_1.pdf]

# Online Continuing Medical Education and the COVID-19 Pandemic

A Survey by Medizin TO GO
(GYN TO GO, NOW TO GO, AINS TO GO, NCH TO GO, OU TO GO, PAED TO GO)

Dear users of Medizin TO GO,
Even prior to the COVID-19 pandemic and relevant increase in value of contactless continuing medical education CME, Medizin TO GO has been providing easy-access, free of charge and independent online medical education. Presently, together with you we are experiencing the digital transformation of the CME and conference environment. By taking this survey, you can assist us to better understand this transformation in addition to improving Medizin TO GO.

Please take 5 minutes to anonymously answer the following questionnaire.
We are very grateful for your support.
Your Medizin TO GO team

*Data protection/Privacy policy according to terms and conditions of SurveyMonkey

**1. Age**

**2. Gender**
- M
- F
- D
- Other

**3. Country of origin**
- Germany
- Austria
- Switzerland
- The Netherlands
- Other European countries
- Outside of Europe

**4. Occupation**
- Physician
- Medical student
- Midwife
- Nurse
- Paramedic
- Physiotherapist
- Other

5. Physicians’ occupation (All other occupations continue with question 7):
- ○ Hospital employee
- ○ Practice employee
- ○ Practitioner/Practice owner
- ○ Other

6. Physicians’ experience level
- ○ Resident
- ○ Board certified
- ○ Attending/Consultant
- ○ Head of department
- ○ Practitioner

7. What resources do you utilize for CME (Multiple answers are feasible)
- ○ Websites
- ○ Scientific journals
- ○ Books
- ○ Face-to-face lectures
- ○ Online lectures
- ○ Colleagues
- ○ I do not attend CME
- ○ Others

8. Privately used Social Media
- ○ Facebook
- ○ Twitter
- ○ Instagram
- ○ Linked-In
- ○ Researchgate
- ○ None
- ○ Others

9. Professionally used Social Media
- ○ Facebook
- ○ Twitter
- ○ Instagram
- ○ Linked-In
- ○ Researchgate
- ○ None
- ○ Other

10. How many face-to-face CME events have you attended yearly?

| | < 5 X | 5 - 10 X | > 10 X |
|---|---|---|---|
| Prior to the pandemic | ○ | ○ | ○ |
| Since the pandemic (March 2020) | ○ | ○ | ○ |

11. Did your user behaviour regarding online CME change since the pandemic?

- ○ Yes, I attended less
- ○ Yes, I attended more
- ○ No

12. What was/were your biggest concern(s) regarding online CME prior to the pandemic? (Multiple answers are feasible)

- ○ Technical issues
- ○ Expenses
- ○ Content
- ○ Dependency e.g. on industry sponsoring
- ○ Data protection / Privacy policy
- ○ Time issue
- ○ No concern

13. What was/were your biggest concern(s) regarding online CME since the pandemic? (Multiple answers are feasible)

- ○ Technical issues
- ○ Expenses
- ○ Content
- ○ Dependency e.g. on industry sponsoring
- ○ Data protection / Privacy policy
- ○ Time issue
- ○ No concern

14. Have you attended online CME events (Webinars) in general, not only TO GO events?

| | Yes | No |
|---|---|---|
| Prior to the pandemic | ○ | ○ |
| Since the pandemic (March 2020) | ○ | ○ |

15. Have you ever attended TO GO online CME events (Webinars)?
(If your answer is No/No, please continue with question 24)

| | Yes | No |
|---|---|---|
| Prior to the pandemic | ○ | ○ |
| Since the pandemic (March 2020) | ○ | ○ |

16. How often yearly have you attended online CME events (Webinars) in general, not only TO GO events?

| | < 5 X | 5 - 10 X | > 10 X |
|---|---|---|---|
| Prior to the pandemic | ○ | ○ | ○ |
| Since the pandemic (March 2020) | ○ | ○ | ○ |

17. How often yearly have you attended TO GO online CME events (Webinars)?

| | < 5 X | 5 - 10 X | > 10 X |
|---|---|---|---|
| Prior to the pandemic | ○ | ○ | ○ |
| Since the pandemic (March 2020) | ○ | ○ | ○ |

18. Which TO GO special field(s) did you attend prior to the pandemic?
- ○ GYN TO GO
- ○ NOW TO GO
- ○ Both

19. Which TO GO special field(s) did you attend since the pandemic? (Multiple answers are feasible)
- ○ GYN TO GO
- ○ NOW TO GO
- ○ AINS TO GO
- ○ NCH TO GO
- ○ OU TO GO
- ○ PAED TO GO

20. Which TO GO special field online CME events do you visit most frequently?
- ○ AINS TO GO
- ○ GYN TO GO
- ○ NCH TO GO
- ○ NOW TO GO
- ○ OU TO GO
- ○ PAED TO GO

21. Which online CME event schedule is more suitable for you to attend?
- ○ Morning events
- ○ Evening events
- ○ Weekend events

22. Do you attend online CME events alone or in a group?
- ○ Alone
- ○ Group
- ○ Both

23. If you attend online CME in a group, on average how many participants take part?

Morning

Evening

Weekend

24. Do you use the CME certification process?
- ○ Yes
- ○ No

25. Do you use the donation option?
- Yes, I donated once
- Yes, I donated multiple times
- No, not yet

26. How satisfied are you with TO GO as an online CME provider? 0 - 100%

27. How important is free of charge online CME in your opinion?
0 - 10 (not at all – very)
0 1 2 3 4 5 6 7 8 9 10

28. How important is independent (from industry) online CME in your opinion?
0 - 10 (not at all – very)
0 1 2 3 4 5 6 7 8 9 10

29. How important is independent (from medical societies) online CME in your opinion?
0 - 10 (not at all – very)
0 1 2 3 4 5 6 7 8 9 10

30. How important is the opportunity to actively participate in online discussions in your opinion?
0 - 10 (not at all – very)
0 1 2 3 4 5 6 7 8 9 10

31. How important is the opportunity to anonymously participate in online discussions in your opinion?
0 - 10 (not at all – very)
0 1 2 3 4 5 6 7 8 9 10

32. How important is it to see the lecturer during online CME in your opinion?
0 - 10 (not at all – very)
0 1 2 3 4 5 6 7 8 9 10

33. What is more appealing in your opinion: (Multiple answers are feasible)
- Events taking place regularly
- Single sporadic events

34. What would you personally prefer to attend regardless of the pandemic:
- Online events
- Face-to-face events
- Both/Blended learning

35. How should the availabilty of online CME be after the pandemic?
- Similar to during the pandemic
- Less available, due to returning face-to-face events

36. The content of online CME should be in your opinion:
Basic knowledge with structured curricula
0 - 10 (not appropriate – fully appropriate)
0 1 2 3 4 5 6 7 8 9 10

37. The content of online CME should be in your opinion:
Special knowledge with focus on specific main topics
0 - 10 (not appropriate – fully appropriate)
0 1 2 3 4 5 6 7 8 9 10

38. The content of online CME should be in your opinion:
Presenting evidence based medicine
0 - 10 (not appropriate – fully appropriate)
0 1 2 3 4 5 6 7 8 9 10

39. The content of online CME should be in your opinion:
Presenting expert opinions e.g. in expert discussions / meet the experts events
0 - 10 (not appropriate – fully appropriate)
0 1 2 3 4 5 6 7 8 9 10

40. Which online CME format is more appealing to you:
- Expert discussions / meet the experts events
- Lectures with subsequent discussion
- Both

41. What duration is optimal for online CME events with subsequent discussion:
- < 1 hour
- 1-2 hours
- 2-3 hours

42. What is your opinion regarding online congresses / conferences?
0 - 10 (not appealing – very appealing)
0 1 2 3 4 5 6 7 8 9 10

43. Would you recommend online CME in general?
0 - 10 (not at all – Fully)
0 1 2 3 4 5 6 7 8 9 10

44. Improvement suggestions / comments / feedback
